# Regression analysis of growth responses to water depth in three wetland plant species

**DOI:** 10.1093/aobpla/pls043

**Published:** 2012-12-18

**Authors:** Brian K. Sorrell, Chris C. Tanner, Hans Brix

**Affiliations:** 1 National Institute of Water and Atmospheric Research, Hamilton, New Zealand; 2 Department of Bioscience, Plant Biology, Aarhus University, Ole Worms Allé 1, DK-8000 Aarhus C, Denmark

## Abstract

**Background and aims:**

Plant species composition in wetlands and on lakeshores often shows dramatic zonation, which is frequently ascribed to differences in flooding tolerance. This study compared the growth responses to water depth of three species (*Phormium tenax, Carex secta* and *Typha orientalis*) differing in depth preferences in wetlands, using non-linear and quantile regression analyses to establish how flooding tolerance can explain field zonation.

**Methodology:**

Plants were established for 8 months in outdoor cultures in waterlogged soil without standing water, and then randomly allocated to water depths from 0 to 0.5 m. Morphological and growth responses to depth were followed for 54 days before harvest, and then analysed by repeated-measures analysis of covariance, and non-linear and quantile regression analysis (QRA), to compare flooding tolerances.

**Principal results:**

Growth responses to depth differed between the three species, and were non-linear. *Phormium tenax* growth decreased rapidly in standing water >0.25 m depth, *C. secta* growth increased initially with depth but then decreased at depths >0.30 m, accompanied by increased shoot height and decreased shoot density, and *T. orientalis* was unaffected by the 0- to 0.50-m depth range. In *P. tenax* the decrease in growth was associated with a decrease in the number of leaves produced per ramet and in *C. secta* the effect of water depth was greatest for the tallest shoots. Allocation patterns were unaffected by depth.

**Conclusions:**

The responses are consistent with the principle that zonation in the field is primarily structured by competition in shallow water and by physiological flooding tolerance in deep water. Regression analyses, especially QRA, proved to be powerful tools in distinguishing genuine phenotypic responses to water depth from non-phenotypic variation due to size and developmental differences.

## Abstract (precis)

Variability in plant flooding tolerance is often associated with differential growth responses to increasing water depth. This study highlights how morphological responses conferring flooding tolerance differ, using non-linear and quantile regression to quantitatively compare flooding-related growth responses of three species.

## Introduction

Plant species zonation is a characteristic feature of water depth gradients in wetland environments and lake shorelines (Seabloom *et al.* 2001; Strayer and Findlay 2010). Zonation develops due to competitive interactions, grazing and disturbance by animals, physical disturbance by water movement and wave action, and differences in physiological flooding tolerance adaptations that support growth in standing water (Keddy 2010). The factors associated with flooding that stress plants in deep water include the limited ability of most wetland species to assimilate inorganic carbon from water (Colmer and Pedersen 2008), light attenuation and its effects on photosynthesis and development (Mommer and Visser 2005), and oxygen deprivation in below-ground rhizomes and roots growing in anaerobic sediments (Bailey-Serres and Voesenek 2008). These abiotic stresses, and the extent to which they can be avoided or tolerated due to ecophysiological adaptations that confer flooding tolerance, become increasingly growth limiting as water depth increases (Sorrell and Hawes 2010). A wide range of morphological, anatomical and biochemical responses that are induced when plants are either waterlogged or submerged have been described over the last 40 years, and shown to contribute to survival and growth in natural wetlands (Colmer and Voesenek 2009; Webb *et al.* 2012).

A notable feature of wetland zonation is the persistence and dominance of helophytes (emergent species with underwater buds) at all depths when standing water is present. Plants in standing water require the same below-ground adaptations for dealing with oxygen deprivation (e.g. aerenchyma formation in roots) as all wetland species growing in permanently anoxic soils, but face the additional challenge of transporting oxygen from the atmosphere to the below-ground tissue through the water, via the shoot aerenchyma. This challenge is not trivial, as the flux of oxygen delivered by gas-phase diffusion decreases rapidly with transport distance (Armstrong *et al.* 1991). Less oxygen delivered by shoots means less below-ground growth (White and Ganf 1998) and, coupled with the much greater fraction of the shoot unable to participate in photosynthetic C assimilation, forces plants to respond to deep water by altering shoot morphology to a smaller number of taller shoots (Vretare *et al*. 2001). These responses are seen even in species such as *Phragmites australis* and *Typha* spp., which have internal convective gas flows that provide much greater internal oxygen flux than diffusion (Armstrong *et al.* 1991), and such responses are strongly linked to depth penetration (Sorrell and Hawes 2010), as plants approach their depth limits. Fewer, taller shoots with depth is an extremely common feature of helophyte depth responses (Webb *et al.* 2012), so quantifying this development can provide valuable insight into how wetland plants become stressed and ultimately killed by standing water.

Many species also respond differently to fluctuating water levels and to sudden increases in water depth than they do to deep but stable water levels. Although growth can be enhanced by fluctuating water levels in fast-growing, phenotypically plastic species such as *Phalaris arundinacea* (Miller and Zedler 2003), many species are less productive in fluctuating than in stable water (Edwards *et al*. 2003; Deegan *et al*. 2007), and most also suffer significantly decreased growth in response to a large, sudden depth increase (Perata *et al*. 2011). Current climate models are predicting a much greater frequency of sudden storm events accompanied by rapid water depth increases in lowland wetland habitats (Zedler 2010), but very few studies have attempted to compare the effects of sudden depth increases on the growth and morphological responses of species adapted to different elevations in shoreline zonation.

Growth responses to environmental stresses often feature non-linear relationships in which variances are highly heterogeneous, especially when confounded with factors other than the stress factor under consideration. In flooding tolerance studies, it can be particularly difficult to distinguish genuine phenotypic responses to depth from non-phenotypic variation associated with size and development (Vretare *et al.* 2001). This study therefore features a regression-based non-categorical comparison of depth responses between three species differing in flooding tolerance, and analysis by non-linear regression and quantile regression analysis (QRA). The benefits of QRA are explored given its recent growing popularity in ecology for detecting functional relationships in data for all portions of a probability distribution, especially when multiple factors affect morphology and biomass (Visser and Sasser 2009). The aim of the study was therefore to explore how various regression techniques can be applied to water depth–plant response data, to provide greater insight into growth responses than categorical analysis of variance (ANOVA)-based experimental designs.

## Methods

### Study species

Three wetland species with well-documented differences in flooding tolerance adaptations, depth preferences and responses to water depth were used. All three species are native to New Zealand, and widespread and common in lowland minerotrophic wetlands (Johnson and Gerbeaux 2004) and have previously had their flooding tolerance adaptations investigated (Brix *et al.* 1992; Sorrell *et al.* 2001). *Phormium tenax* is a facultative wetland species that thrives under intermittent waterlogging and flooding, but has reduced growth and greater mortality in permanent standing water. *Carex secta* is a large tussock sedge that tolerates prolonged waterlogging and flooding, albeit with conspicuously reduced growth in permanent standing water. *Typha orientalis* is an obligate wetland helophyte indefinitely tolerant of waterlogging and of flooding to water depths >1 m (Sorrell and Hawes 2010). All three species have aerenchymatous shoots and roots, but root porosity is much lower (<20%) in *P. tenax* than the other two species (ca. 40–50% porosity; Sorrell *et al*. 2001). There is also well-developed internal pressurization and convective gas flow in *T. orientalis* (Brix *et al*. 1992), but no pressurization or flow in either *C. secta* or *P. tenax* (Sorrell *et al*. 2001).

### Experimental design

One-month-old seedlings of the three species were obtained from a specialist native plant nursery (Motukarara Conservation Nursery, Christchurch, New Zealand) and established as monospecific experimental cultures in a common garden design at the Silverstream Research Facility, 15 km north of Christchurch, New Zealand (43°33′S, 172°47′E). Each culture was established from a single seedling planted in the centre of a plastic crate (0.5 × 1.0 × 0.5 m) containing 0.20 m^3^ floodplain soil (total nitrogen = 700 mg N kg^−1^ dry weight (DW), total phosphorus = 400 mg P kg^−1^ DW), initially flooded to the surface in a concrete runway under a continuous through-flow of water from the adjacent spring-fed Kaiapoi River (constant temperature = 12 °C). The soils were flooded for 5 months prior to planting to ensure wetland soil anoxia and reducing conditions, which was determined from redox potentials (Eh) measured with permanently installed (0.25 m depth) welded platinum wire electrodes (Faulkner *et al*. 1989), using a saturated Ag/AgCl reference electrode with +199 mV added to correct to Eh readings (Armstrong *et al*. 1985). All populations were then allowed to establish at the same water depth (flooded to soil surface, i.e. water depth = 0 m) for 8 months over the austral autumn–winter–spring months of May–November. At this time the mean Eh value in the 27 cultures was 185 ± 16 mV, typical of permanently waterlogged, anoxic and moderately wetland soils (Faulkner *et al*. 1989). The experiment was then initiated by randomly allocating the crates to nine new depths between 0 and 0.5 m for the nine cultures of each species.

### Plant measurements

#### Non-destructive shoot morphology

Non-destructive measurements were performed immediately prior to flooding and then repeated 16, 27, 40 and 54 days after flooding during the spring–summer growth season (November–January) before the experiment was harvested. This involved tagging and monitoring the development, maturation and senescence of shoots, following responses of the morphological parameters listed in Table 1 to flooding. Redox potential measurements were also repeated at each sampling date.

**Table 1:** **Summary of repeated-measures ANCOVAs on sequential morphological measurements for *P. tenax, C. secta* and *T. orientalis*.** The repeated measure (Time) based on measurements on Days 0, 16, 27, 40 and 54 after the establishment of new depths, with depth of cultures as the covariate. *F* values shown with degrees of freedom are in parentheses. Positive (+) and negative (−) main effects of time and depth are shown after significant *P* values (denoted by bold text). Variables that did not satisfy homogeneity assumptions (*C. secta*, number of live shoots; *T. orientalis*, number of ramets) were log-transformed before analysis.

Parameter	Time		Depth (covariate)		Depth × Time	
	*F*_(1,7)_	*P* (effect)	*F*_(4,4)_	*P* (effect)	*F*_(4,4)_	*P* (effect)
*Phormium tenax*						
Number of ramets	3.11	0.15	3.9	0.089	2.56	0.19
Cumulative leaf length	47.40	**0.0013 (+)**	11.0	**0.013 (−)**	19.53	**0.0069**
Mean live leaf length	0.71	0.63	0.11	0.75	0.83	0.57
Number of live leaves	0.53	0.17	13.9	**0.041 (−)**	0.54	0.72
Number of dead leaves	1.09	0.41	3.2	0.12	1.29	0.41
Tallest live leaf	0.28	0.88	1.53	0.26	0.30	0.87
*Carex secta*						
Number of live shoots	32.71	**0.0026 (+)**	22.0	**0.0022 (−)**	17.67	**0.0083**
Number of dead shoots	0.21	0.92	0.45	0.52	0.37	0.82
Mean shoot height	30.26	**0.015 (+)**	8.67	**0.0001 (+)**	6.42	0.038
*Typha orientalis*						
Number of ramets	2.00	0.26	2.53	0.16	0.96	0.51
Cumulative leaf length	4.17	**0.040 (+)**	2.37	0.17	1.47	0.36
Mean live leaf length	1.30	0.40	0.77	0.41	0.36	0.83
Number of live leaves	7.41	**0.025 (+)**	2.26	0.17	1.47	0.36
Number of dead leaves	0.45	0.77	1.28	0.30	0.16	0.95
Tallest live leaf	0.79	0.60	0.14	0.72	0.46	0.26

#### Harvest procedure

On Day 54, plants were removed from boxes by gentle washing of the substrate free from roots and rhizomes, and were separated into live (green) and dead (yellow/brown) tissue and biomass fractions (leaves, shoots, shoot base, rhizome, roots) within individual ramets. Material was dried at 70 °C for 48 h before weighing.

### Data analysis

Data analyses were based on using regression approaches to determine effects of depth as a continuous independent variable on growth and morphology. The sequential non-destructive morphological measurements (listed in Table 1) and Eh data were analysed using a repeated-measures analysis of covariance (ANCOVA), with the repeated measure ‘time’ and depth as a covariate to determine the extent to which depth affected development over time. Data were examined for homogeneity of variances (Bartlett's test, *P* < 0.05) and log_10_(*x* + 1) transformed if they failed to satisfy homogeneity assumptions. Final harvest data were explored with linear and non-linear regression including logistic sigmoid and logistic with hormesis approaches (Stephenson *et al*. 2000), with the models selected being those with lowest variance of residuals. The residuals were also examined for homogeneity of variance over the depth gradient using White's test (White 1980) and always satisfied homogeneity assumptions. Further analysis of biomass and morphology depth responses involved least-squares regression (LSR) and QRA of morphological and biomass parameters relevant for each species (Table 2). The QRA provides estimates of the maximum growth response vs. depth, independent of data outliers and increasing/decreasing variance homogeneity with depth (Cade and Noon 2003). The significance of quantile regression slopes was calculated with the regression rank score inversion method (Koenker and d'Orey 1987). Data were not transformed for QRA as it is a non-parametric test that makes no assumptions regarding normality of distribution or variance homogeneity. Statistical analyses were performed using JMP 9.0.0 for all repeated-measures ANCOVA and linear regression analyses, and the SAS quantile regression add-in for JMP for quantile regressions.

**Table 2:** **Summary of the QRA for *P. tenax* and *T. orientalis*.** All models are single-parameter quantile fits with depth as the independent variable *x* and determined at *τ* = 0.90 and *τ* = 0.95. DM, dry mass. Models are functions of the measured dependent variables vs. depth, with *t* and *P* statistics. All quantile regressions with *P* ≤ 0.10 are shown; regressions with *P* > 0.10 are identified as n.s. (not significant).

Dependent variable		*τ* = 0.90		*τ* = 0.95	
	*N*	Model	*t* (*P*)	Model	*t* (*P*)
*Phormium tenax*					
Live leaf DM per ramet (g)	44	22.5–32.9*x*	1.38 (0.17)	28.3–46.3*x*	2.94 (<0.005)
Live leaf length per ramet (m)	44	2.23–1.97*x*	1.35 (0.08)	2.45–1.90*x*	1.46 (0.04)
Senescent leaf DM per ramet (g)	44	2.90–5.44*x*	3.31 (<0.001)	4.04–6.68*x*	2.05 (0.04)
Number of leaves per ramet	44	5.32–2.94*x*	1.84 (0.06)	6.12–4.00*x*	1.92 (0.04)
Individual leaf lengths (m)	137	n.s.	0.09 (0.93)	n.s.	0.35 (0.73)
Individual leaf widths (mm)	137	17.8 + 20.0*x*	2.02 (0.04)	n.s.	1.35 (0.17)
*Typha orientalis*					
Live leaf DM per ramet (g)	41	n.s.	0.75 (0.45)	n.s.	0.79 (0.43)
Live leaf length per ramet (m)	41	n.s.	0.65 (0.52)	n.s.	1.14 (0.25)
Senescent leaf DM per ramet (g)	41	n.s.	1.17 (0.12)	n.s.	1.27 (0.20)
Number of leaves per ramet	41	n.s.	0.26 (0.79)	n.s.	0.62 (0.54)
Individual leaf lengths (m)	170	n.s.	0.30 (0.50)	n.s.	0.03 (0.90)
Individual leaf widths (mm)	170	n.s.	0.01 (0.95)	n.s.	0.01 (0.95)

## Results

### Survival and morphological responses

All three species had 100% survival in their nine cultures at all water depths. Soil Eh readings remained stable between +115 and +198 mV throughout the experiment and were unaffected by flooding depth (*P* = 0.65).

**Fig. 1:**
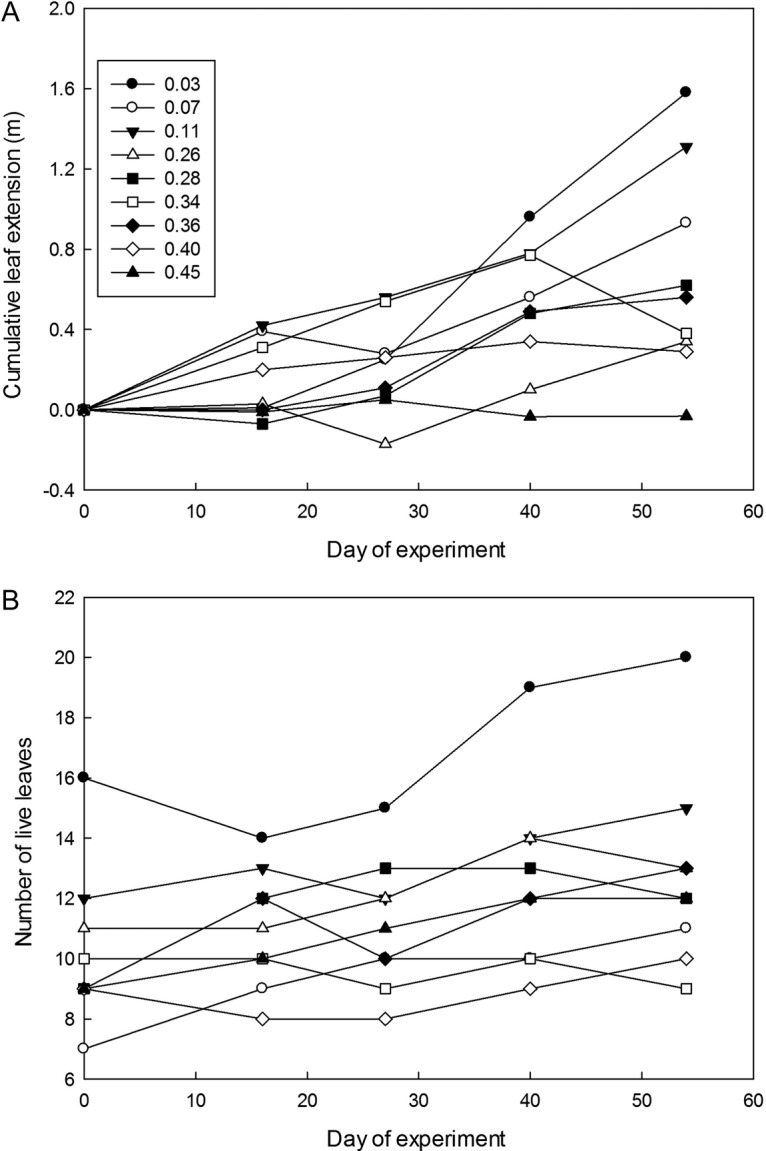
**Effect of water depth on (A) cumulative leaf extension and (B) change in number of live leaves of *P. tenax*.** Each line represents one of the nine cultures at randomly allocated flooding depths (depths in metres) shown in the key in (A). Negative values in (A) occur when cultures had less total leaf length than at the start of the flooding treatment. See Table 1 for ANCOVA analysis.

Morphological responses to depth over time were revealed by repeated-measures ANCOVA (Table 1), and sensitivity to water depth differed between species. In *P. tenax*, the number of ramets remained unchanged, as no new ramets were produced during the 54 days of the experiment and growth was limited to increasing size of existing ramets. The increase in total leaf length was strongly affected by depth (Fig. 1A), with much less leaf extension as depth increased, and the significant depth × time interaction indicating how the negative effect of depth became stronger over time (Table 1). The mean leaf length within cultures remained unchanged, due to the appearance of new young leaves counteracting the increasing length of existing leaves. The number of live and dead leaves nevertheless did not change significantly over time, because there was considerable turnover and abscission of older leaves during the experiment, and the number of live leaves ultimately became lower in deeper water as deeper cultures failed to produce as many new leaves as shallower ones (Table 1, Fig. 1B).

**Fig. 2:**
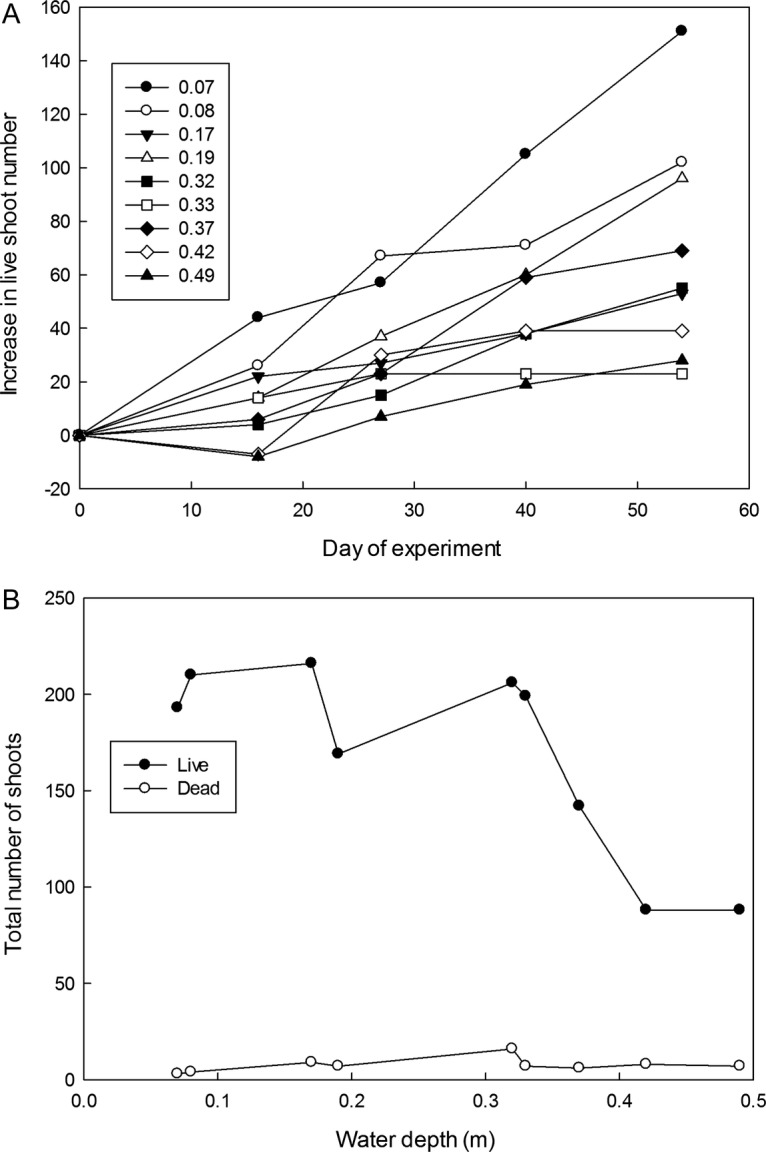
**Effect of water depth on (A) cumulative increase in shoot number and (B) final total shoot number of *C. secta*.** In (A) each line represents one of the nine cultures at randomly allocated flooding depths (depths in metres shown in the key). Negative values in (A) occur when cultures had fewer shoots than at the start of the flooding treatment. Data in (B) are total number of live and dead shoots in each culture. See Table 1 for ANCOVA analysis.

New shoot production continued rapidly in *C. secta*, but there was a strong negative effect of depth on shoot numbers with much less new shoot production in deeper water (Table 1, Fig. 2A). Shoot heights increased at all water depths in this species, but the height increases were much greater in deeper water (Table 1). The overall response of *C. secta* was therefore a shift to taller but fewer shoots in response to depth (Table 1, Fig. 2B). Numbers of dead shoots remained low and did not change with time or depth (Fig. 2B); the depth effects were a consequence of fewer new shoots being produced rather than greater senescence. In contrast to *C. secta*, no significant depth effects were detected on *T. orientalis* morphology, although there was substantial growth at all depths, evident as increasing numbers of leaves and hence increasing total leaf length (Table 1). Table 1 also reveals that no new ramets were formed in this species after the flooding treatments were imposed, nor did the maximum height of the ramets or the mean leaf length increase.

### Final biomass and morphology

**Fig. 3:**
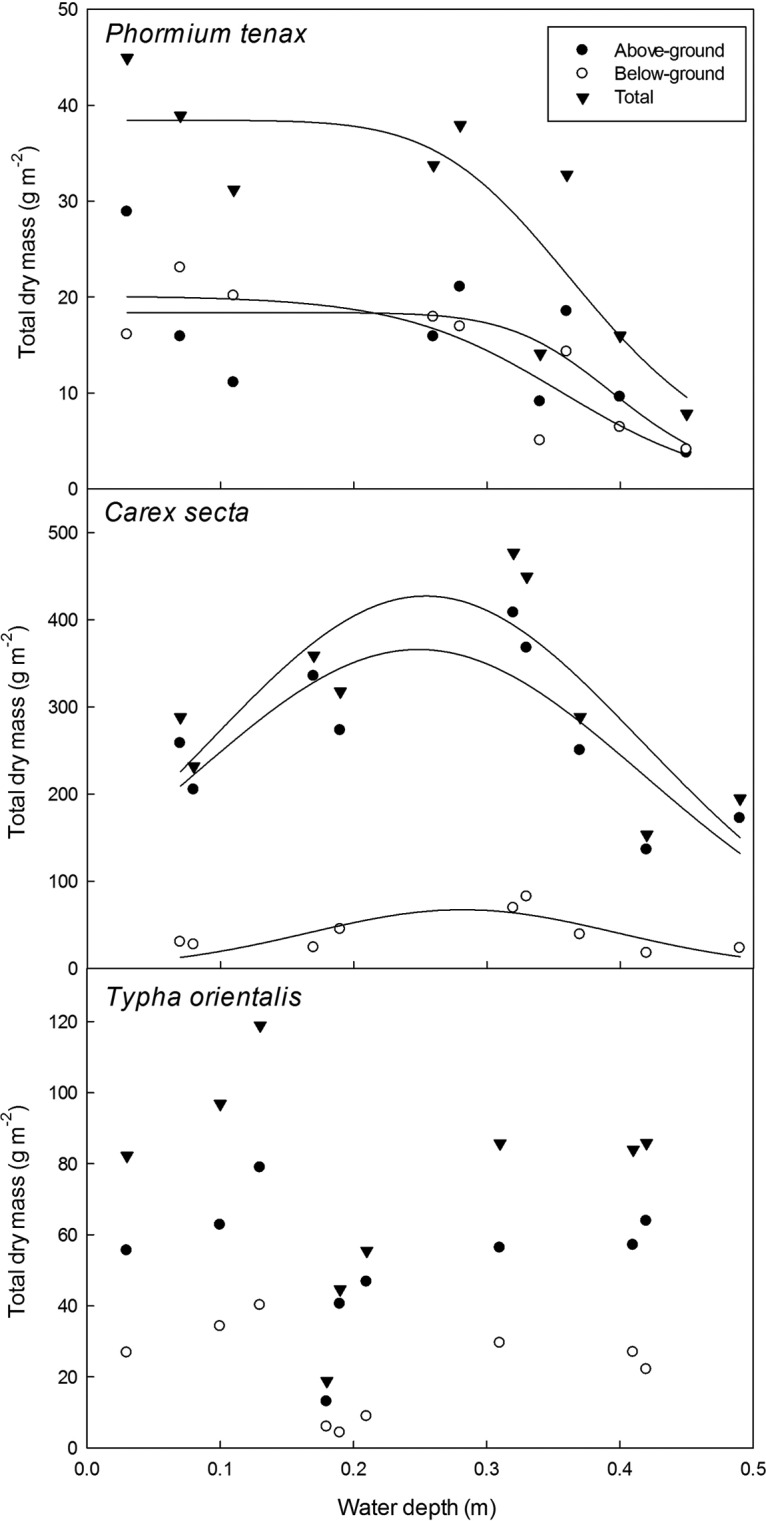
**Effect of water depth on total dry mass in the nine cultures of each species.** Each point is the sum of all material in each of the nine cultures of each species after harvest at the end of the experiment (54 days). Non-linear regression models are curves of best fit for the three species (models with lowest variance of residuals). For *P. tenax*, the sigmoidal logistical model described the depth response, for *C. secta* a logistic model with hormesis best described the response, and for *T. orientalis* there was no significant effect of depth on biomass according to any models.

Water depth responses of total live biomass for the nine cultures of each species are shown in Fig. 3. A sigmoidal logistical model successfully described the decrease in above-ground mass with depth for *P. tenax*, whereas below-ground biomass decreased linearly with depth; the overall depth response for total biomass was also logistic. As both above- and below-ground biomass decreased with depth, there were no significant effects according to any model of depth on below : above ratios, leaf mass ratio or root mass ratio. *Carex secta*, in contrast, showed greatest biomass at intermediate depths, and the best model for its depth response was a logistic–hormesis model, for both above- and below-ground tissue. As with *P. tenax*, there was no significant effect of depth on tissue allocation patterns, as depth affected leaves, shoots and roots similarly. In *T. orientalis* there were no significant trends in any biomass parameters with depth. In all three species, dead biomass was always <15% of the total biomass and there was no significant effect of depth on the amount of dead biomass or the proportion of biomass consisting of dead tissue.

**Fig. 4:**
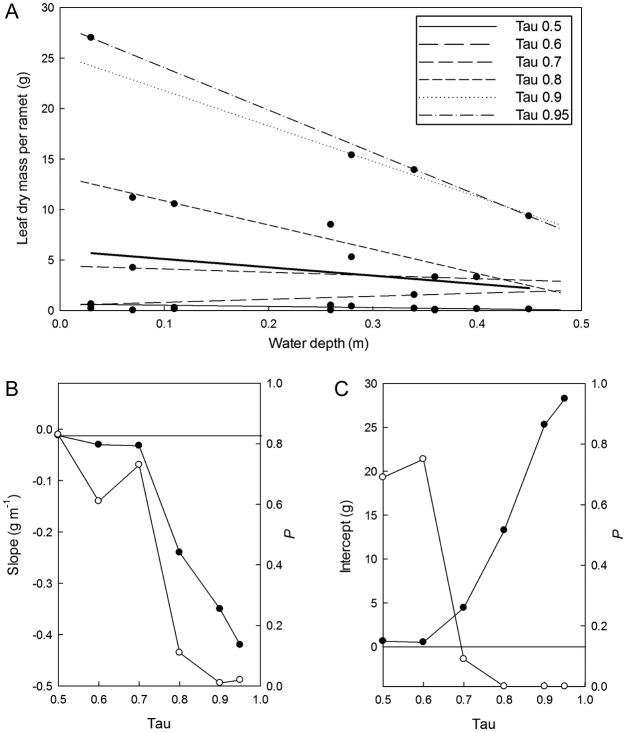
**Effect of water depth on dry mass at final harvest of individual leaves of *P. tenax*.** (A) Plot of leaf dry mass (*n* = 44) at different depths analysed by LSR (bold line) and with 0.50, 0.60, 0.70, 0.80, 0.90 and 0.95 quantile estimates of the depth vs. dry mass relationship. (B) and (C) Functions of slope and intercept vs. *τ* of the quantile regression lines, and significance level (*P*, open circles). See Table 2 for QRA statistics.

Figure 4 provides a detailed example of QRA application to one growth parameter (dry mass of individual ramets of *P. tenax*). In Fig. 4A the scatter of leaf dry mass results in a non-significant (*r*^2^ = 0.04, *P* = 0.31) LSR of depth vs. dry mass for the leaves. Fitting a range of QRA lines with increasing *τ* from 0.5 to 0.95 identified a significant linear depth vs. dry mass relationship at the upper 10% (≥ 90% percentile, *P* ≤ 0.05) of the leaves (Fig. 4B), and an intercept that differed significantly from 0 for the upper 20% of the leaves (Fig. 4C). Quantile regression analysis can thereby exclude younger leaves that have yet to respond to water depth from the analysis.

**Fig. 5:**
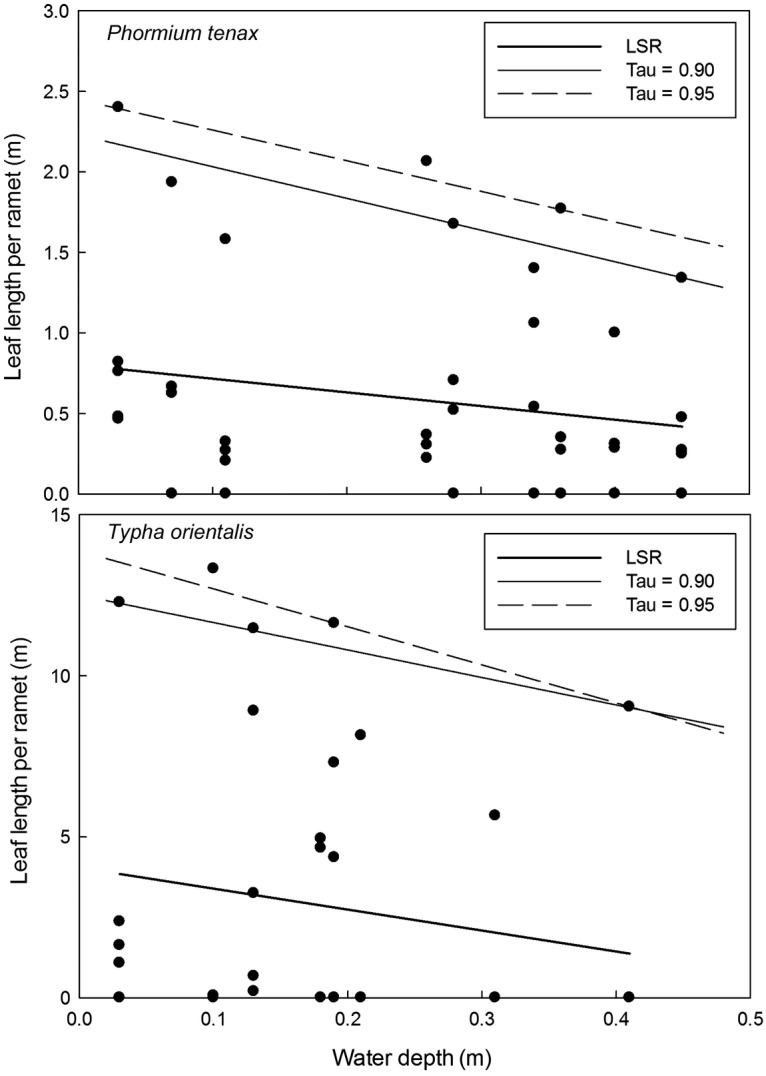
**Effect of water depth on the total leaf length of ramets of *P. tenax* and *T. orientalis.*** Plots of total leaf length for individual ramets (*n* = 44 *P. tenax* ramets, *n* = 40 T*. orientalis* ramets) vs. depth analysed by LSR (bold line) and quantile regression (with *τ* = 0.90 and 0.95). The LSR is not significant for either species (*P* = 0.21 for *P. tenax*, *P* = 0.28 for *T. orientalis*) See Table 2 for QRA statistics.

The effect of water depth on leaf length for the two species in which tall, linear leaves comprise most of the above-ground biomass (*P. tenax* and *T. orientalis*) is shown in Fig. 5. In *P. tenax*, QRA provided significant estimates of the effects of depth on leaf length that are not captured by LSR. At *τ* = 0.90 the quantile regression was significant at *P* < 0.10, increasing to *P* < 0.05 with *τ* = 0.95. The 95% regression quantile estimate provided the strongest negative linear relationship between depth and both dry mass and length of *P. tenax* leaves, with the lowest *P* and lowest variance estimates. The significant effects of depth on *P. tenax* leaves can be contrasted with *T. orientalis*, in which neither LSR nor QRA produced significant depth effects. Although the slopes of the upper quantile regressions were also negative in this species (Fig. 5), they were never significant, even at high *τ* values.

The QRA of depth responses of morphological parameters is summarized for *P. tenax* and *T. orientalis* in Table 2. It shows the effect of depth on *P. tenax* is to reduce numbers of leaves per ramet, and thereby total leaf length and both live and dead dry mass, but not the length or width of individual leaves. Together with the time series in Fig. 1, this reveals how the sudden depth increase imposed by the experiment had little effect on the pre-existing leaves, which the plants were able to maintain, but inhibited new leaf growth, especially at depths ca. >0.2 m (Fig. 1, cf. Figs 3–5). This is in contrast to *T. orientalis*, in which growth remained unaffected over the 0.5 m depth increase range of this experiment, with no significant regressions with either LSR or at any quantile level (Table 2).

**Fig. 6:**
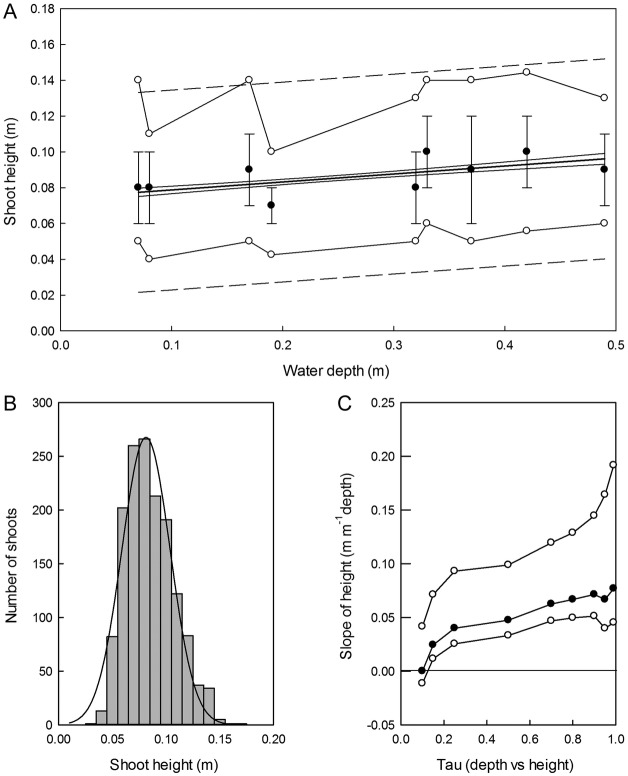
**Effect of water depth on the shoot height of *C. secta*.** (A) Least-squares regression analysis (bold line) with 95% confidence intervals (solid lines) and prediction intervals (dashed lines). Closed circles are mean numbers of shoots at each depth (±1 standard deviation), open circles show upper and lower 95% quantiles of data identified by QRA (LSR = 0.064 + 0.011 × depth, *r*^2^ = 0.07, *P* < 0.0001). (B) Histogram of shoot heights from all *Carex* plants in the experiment (bars are 0.01 m intervals), with fitted normal distribution. (C) Quantile regression analysis showing change in slope of quantile regression estimates (filled circles) at increasing *τ* with upper and lower 95% confidence intervals (open circles). The slope estimates are not significant at *τ* < 0.15, but are always significant (*P* < 0.0001) at *τ* ≥ 0.15. See Table 2 for QRA statistics.

*Carex secta* had produced large numbers of shoots at all depths by the end of the experiment, with a total of 1498 shoots across the entire depth gradient, and numbers of shoots in the nine cultures varying from 88 to 216. The large number of shoots meant both LSR and QRA were able to produce highly significant depth–height relationships, but QRA was able to explain more of the variation than LSR. The LSR in Fig. 6A has narrow confidence intervals and is significant at *P* < 0.0001 because of the large sample size, but has *r*^2^ = 0.07, indicating that depth alone explains little of the overall variation in shoot height, and upper and lower 95% quantiles and prediction intervals reveal a wide range of shoot heights at any given depth. As the overall distribution of shoot heights across all depths at the end of the experiment was approximately normal (Fig. 6B), both parametric (LSR) and non-parametric (QRA) models fitted the data closely, but the 50% QRA (i.e. median) response (height = 0.067 + 0.047 × depth) had a steeper slope than the LSR despite a similar intercept (cf. Fig. 6A). The slopes of the QRA lines increased with increasing *τ* (Fig. 6C), although the upper quantiles (>*τ* = 0.80) had similar slopes. In Fig. 6C, the slope response becomes significant at *τ* ≥ 0.15, and the upper confidence interval increases at high *τ* values, which demonstrates that while most shoots increase height in deeper water, the response is greatest in the tallest shoots.

## Discussion

The present study of emergent plant development after a sudden increase in water depth revealed that growth and morphological responses differed between the three species over the 0.5 m depth gradient, in accordance with their documented field zonation (Johnson and Gerbeaux 2004) and flooding tolerance adaptations (Sorrell *et al*. 2001). Applying the same depth range for all three species can highlight differences in species' responses that suit them to a particular water regime (Deegan *et al.* 2007), and treating depth as a continuous rather than a categorical variable revealed its non-linear effect on morphological and growth responses. Although the greatest water depth applied in this study was not lethal for any of the species over the timeframe of this experiment, the repeated-measures ANOVA and regressions clearly allowed their flooding tolerances to be contrasted. Furthermore, the non-linear regression approach allowed depth thresholds beyond which the less flood-tolerant species were negatively affected by standing water to be clearly identified (ca. 0.25 and 0.30 m depth for *P. tenax* and *C. secta*, respectively), and can also be extrapolated to allow predictions of maximum lethal depths. Regressions were also successful in identifying which growth features were *not* affected by flooding, especially allocation patterns, as expressed by parameters such as below : above-ground ratios, and leaf and root mass allocation. Flooding increases shoot : root ratios and allocation to leaves in many wetland taxa (Kercher and Zedler 2004), although this is most often observed in small, young plants, and the lack of response of such parameters in our experiment is likely to be a consequence of our experimental units being larger, well-established cultures where some of the pre-existing biomass did not respond to the depth increase. Many flooding tolerance studies are carried out using relatively small plants, and this approach has yielded much valuable information about growth responses, especially during establishment (e.g. Smith and Brock 2007; Fraser and Miletti 2008; Banach *et al*. 2009). Our study highlights how larger, established cultures can provide alternative information that may be more relevant to field responses of pre-existing vegetation. A disadvantage of using larger experimental units is that often, less replication is possible due to practical limitations; the regression approach is particularly valuable in such experiments as it does not rely on replicated units at an individual depth. Instead, it can establish significant responses by taking a population approach to ramets and shoots over the entire depth range studied.

A clear qualitative difference in the nature of the flooding stress is evident when comparing regressions in the three species: reduced growth and failure to elongate above-ground leaves in *P. tenax*, reduced growth offset by shoot elongation, particularly of the tallest pre-existing shoots, in *C. secta*, and little effect of depth on *T. orientalis. Typha* species are among the deepest growing of helophytes (Sorrell and Hawes 2010), and the expectation in this study was that the maximum depth of 0.5 m would not cause a flooding-related stress response in *T. orientalis*, in contrast to *P. tenax* and *C. secta. Typha* species differ in their depth responses, but most initially increase shoot length and biomass with depth, and then become stressed by standing water when depth exceeds ca. 0.9–1.0 m (Grace 1989; Inoue and Tsuchiya 2009; Miao and Zou 2012). *Typha orientalis* has received less attention than many other *Typha* species, but is capable of accumulating very high biomass in the field in environments ranging from waterlogged soil without standing water (Pegman and Ogden 2005) to water depths up to 1.0 m (Froend and McComb 1994; Inoue and Tsuchiya 2009). In controlled experiments, it appears to grow equally well in waterlogged soils with or without standing water (Sorrell *et al.* 2001; this study), unlike some other *Typha* species which appear to grow better in standing water than waterlogged soil (Grace 1989).

The ability of *T. orientalis* and other *Typha* spp. to dominate wetland vegetation at depths of 0.5–2.0 m is strongly associated with their development of convective gas flow, as very few species lacking convective flow can persist in water >0.5 m depth (Vretare Strand 2002; Sorrell and Hawes 2010). *Phorium tenax* and *C. secta* do not have convective flow (Sorrell *et al*. 2001), and lack of flow is implicated as the explanation for the rapid decrease in growth they suffered in this experiment as water depth increased above 0.25–0.3 m. Convective flow greatly increases internal oxygen concentrations and below-ground growth over the long distances that oxygen must be transported when there is standing water (Armstrong *et al.* 1991; White and Ganf 1998). Aeration differences may also explain the greater flooding tolerance of *C. secta* than *P. tenax*. Both species have relatively limited shoot aerenchyma development, but root porosity is much greater in the former than in the latter (Sorrell *et al*. 2001), and a greater root aeration capacity may allow oxygen deprivation caused by the limited shoot oxygen supply to be avoided more in *C. secta* than in *P. tenax*.

The wide variety of growth forms present in most wetland and shoreline vegetation is an important consideration complicating the understanding of depth effects. Rhizomatous perennials with cylindrical leafless culms (common in genera such as *Eleocharis*, *Juncus* and *Schoenoplectus*), broad-leaved species such as *Typha* spp. and narrower-leaved genera such as *Carex* that may be variously tussock- or sward-forming have different morphological constraints controlling their depth responses, and hence may be difficult to compare directly in growth experiments. The three species in this study differed considerably in their morphology, but relative responses of specific morphological parameters relevant for each taxon to similar depth ranges were able to distinguish their depth preferences and tolerances. Like most wetland monocots, all three species are clonal and QRA could be applied on a population basis to shoots produced across the depth gradient. The great benefit of QRA is that the change in slope and its significance with increasing *τ* identifies which subset of the population of shoots is being affected by depth, independently of any differences in shoot length or weight that are not depth related. In plant development, size itself and ontogenetic change can confound attempts to link apparent growth responses to specific environmental factors (Gedroc *et al*. 1996; Vretare *et al.* 2001), and with QRA the upper and lower quantiles can provide more relevant information than a conventional regression (Cade *et al*. 1999). During development, newly formed shoots and leaves may show less response to environmental stressors than older material that has been exposed to the stressor longer. Quantile regression analysis therefore provides a more nuanced insight into flooding responses than LSR, including responses identified here such as depth affecting numbers of leaves per ramet rather than length of individual leaves in *P. tenax*, and the tallest existing shoots in *C. secta* being those most positively affected by increased depth.

An increase in shoot length accompanied by a decrease in shoot density is perhaps the most ubiquitous of all growth responses distinguishing flood-tolerant from flood-sensitive species. It is a particularly characteristic response of the deepest-growing helophytes in wetland communities (Edwards *et al*. 2003; Macek *et al*. 2006; Smith and Brock 2007), and is usually interpreted as a response that maintains gas exchange with the atmosphere (Vretare *et al.* 2001; Deegan *et al*. 2007). It also improves light penetration to the underwater tissues, which may assist those species able to photosynthesize under water (Colmer and Pedersen 2008). This was the most consistent response to increased water depth in a recent meta-analysis by Webb *et al*. (2012), whereas biomass and allocation responses to flooding were more variable. The inability of *P. tenax* to adjust morphologically to depth, in contrast to the depth accommodation response in *C. secta* and robust growth at all depths in *T. orientalis*, is consistent with the depths at which the three species are observed in the field (Johnson and Gerbeaux 2004). However, all three species grew very well in waterlogged soil without flooding, supporting the contention by Keddy (2010) that wetland plants tolerate extremes of flooding but do not physiologically require them, and that the apparent requirement for flooding in these species is more ecological than physiological. Keddy (2010) further suggests that most wetland species have broader physiological tolerance to flooding than the depths at which they occur in the field, zonation therefore being driven by competition in shallow water and physiological tolerance in deeper water (Grace 1989; Lenssen *et al*. 1999; Jung *et al.* 2009). The three species in this study fit this model; even *C. secta* which, despite having better growth at 30 cm than at 0 cm depth, rapidly decreased growth at greater depths. Preference for waterlogged soil or very shallow water is characteristic of a number of other clonal wetland monocots (Insausti *et al*. 2001; Bakker *et al*. 2007; Banach *et al*. 2009). Species zonation and vegetation composition in several recent studies (Miller and Zedler 2003; Bakker *et al*. 2007; Raulings *et al*. 2010) also support the flooding tolerance–competition depth gradient proposed by Keddy (2010), which is also consistent with broader competition–stress models in plant communities (Grime *et al.* 1988).

## Conclusions and forward look

The responses to flooding depth of these three species differ both quantitatively and qualitatively, and responses are non-linear. The shallow species *P. tenax* has reduced growth at depths >0.25 m, predominantly associated with lower leaf production rather than any change in numbers of ramets produced; the intermediate species *C. secta* displays a depth accommodation response with increasing height and biomass up to 0.30 m depth, but dramatically reduced shoot production and reduced growth at >0.30 m depth; the deep species *T. orientalis* is unaffected by water depths from 0 to 0.5 m. All three species grew well in waterlogged soil without standing water, supporting the principle that competition is the major factor driving zonation in shallow water but physiological tolerance controls zonation in deep water (Lenssen *et al.* 1999; Jung *et al.* 2009). The depth accommodation response of *C. secta*, which also becomes apparent in *Typha* spp. at depths >0.7 m (Grace 1989; Squires and van der Valk 1992), demonstrates that phenotypic plasticity (i.e. the production of multiple phenotypes from a single genotype, depending on environmental conditions; Miner *et al*. 2005) is an important functional strategy for flood-tolerant wetland plants (Vretare *et al*. 2001). We suggest that the regression methods used in our study, especially QRA, are valuable in distinguishing genuine plasticity from non-plastic (i.e. developmental or size-induced) variation (Vretare *et al*. 2001), and that accurate assessments of plasticity need to be incorporated further in interpretation of wetland flooding tolerance experiments, given the increasing awareness of the role that phenotypic plasticity plays in structuring both abiotic responses and community structure (Valladeres *et al*. 2007).

## Sources of funding

The New Zealand Ministry of Science and Innovation provided funding for this project (Contract C09X1002) and the Danish Council for Independent Research—Natural Sciences (Project Number 272-07-0633) funded the research visit of H.B. to New Zealand.

## Contributions by the authors

B.K.S. undertook the experimental work; all authors contributed to the planning of the research and to the manuscript.

## Conflict of interest statement

None declared.
